# 

**Published:** 1864-12

**Authors:** 


### INDEX TO VOLUME V.

Amenorrhœa, Treatment of. By H. R. Storer, M.D., 34
Anæsthetic, New (or Old), 55
Andrews, E., on Epidemic Erysipelas, &c., 17
" on Nitrous Oxide as an Anæsthetic, 78
" on Surgical Pocket Cases, 129
" on Improved Methods of Counter-Extension, 207
" Report on Surgery, 541
Animal Poisons as Causes of Disease, &c., 144
Asthma, its Pathology and Treatment, 179, 570
Association, American Medical, 183, 430, 653
Address, Valedictory. By Henry Wing, M.D., 193
Abscess of the Gall-bladder. By Henry M. Lyman, M.D., 210
American Medical Association, Transactions of, 244, 248
Annual Report of Indiana Hospital for Insane, 245
" " of New York State Lunatic Asylum, 245
Acknowledgment, 247
Atropine, New Mode of Application, 291
Anæsthesia from Chloroform Prolonged, 304
Aphasia, M. Trousseau on, 316, 654
Addison, R. S., on Hydrophobia, 345
Amerman, George K., on Surgical Cases, 458
Animalculæ of Typhoid Fever, 462
Alcohol and Tobacco, 514
Ambulance, A New One, 587
Allen, J. A., on Spotted Fever, &c. 593
Abbott, N. W., on Conservative Surgery, &c., 609

Page.
Azulene of Patchouly, 122
Albuminaria in Children, Treatment of, 701
Arteria Innominata, Tied, 714, 716
Bright's Disease of Kidney, &c., 71
Brain of a Bushwoman, 100
Black-Leg, Disease termed such among Lumbermen, 102
Belladonna, in Cases of Burns, 104
Books and Pamphlets, 107, 443, 650
British Army, Health in, 124
Births, Proportion of to Population, 124, 716
Bartlett, John, on Bright's Disease, &c., 71
" " on Cerebro-Spinal Meningitis, 395
Bile, Source and Constitution of. By T. Antisell, 162
Braithewaite's Retrospect, 180
Bromine, Formula for Solution of, 188, 561
Bladder, Subpubic Puncture of, 310
Bogue, E. A., on Cleft Palate, &c., 379
Bromine in Dysentery and Diarrhœa, 561, 657
Crist, D. C., on Malformation of Fœtus, 13
Cases in Practice. By Hiram Wanzer, M. D., 21, 278, 410, 612
Chicago Medical Journal, 58
Chicago Medical College Commencement, &c., 107, 182, 300, 586
Chair of Chemistry at Berlin and Bonn, 110
Catalysis, as a Chemical and Physiological Process, &c., 132
Collodion with Glycerine in Erysipelas, &c., 189
Counter-Extension by Improved Methods, 207

Page.
Chicago Medical Society,...246, 586, 706
Common Carotid, Ligation of,...... 269
Calabar Bean. By Christison,...... 283
Cerebro-Spinal Meningitis,..........
26, 81, 296, 395, 403, 405, 593
Cow-Pox, its Origin and Nature,... 813
Cleft-Palate, Remedies for,.......... 379
Constipation, Obstinate,.........381, 654
Cinchona Plant in Jamaica,......... 444
Conservative Surgery—Resections, 609
Cote, W. N., Letters,.............184, 646
Catheterism of Duodenum, &c.,..... 653
Correspondence from New York,... 670
Davis, N. S., Clinical Cases and Reports, ..............................
26, 144, 321, 449, 561, 616, 665

Drought in West Indies,.............. 106
Deaths of Physicians,......119, 246, 462
Disease, New,.......................... 238
Dental Journal, The People's,...... 247
Dysentery, Treatment of by Ipecac, 250
Diphtheria, ..................273, 349, 650
Diabetes, Diet in,..................... 310
Digitalis in the Treatment of Epilepsy, ................................ 315
Disulphite of Quinine as a Poison, 316, 587
Dreaming and Somnambulism,..... 422
Dietary in Disease,.................... 457
Diarrhœa, Chronic,.................... 561
Degeneration and Regeneration of Nerves,................................ 704

Erysipelas, Epidemic in Chicago; Causes of,..........................17, 220
Education, Medical, in France, &c., 171, 298, 299
Ear, Diseases of,....................... 298
Epidemic Diseases, Partial Arrest of, ..................................... 311
Eye, Diseases of,..................385, 651
Esculapian Society, Semi-Annual Meeting of,........................... 442
Essays, Military, Medical, and Surgical,.........................514, 584
Explanations,......................517, 587
Edgar, W. S., on Camp Typhoid Fever, ................................ 65

Fever, Typhoid,.....................65, 462
" Rheumatic, Treatment of,.. 226
" Puerperal, Report on,........ 529
" Spotted,.......................81, 593
Fractures, Ununited, New Operation for,.................................. 416
Fever, Yellow, at Newbern,......... 713

Page.
Fore-arm Wrenched off in Reduction of Dislocation,................ 589
Fillmore's Coagula of the Heart,... 715
Fitch, T. D., on Opium and Quinine,...................................... 661

Galactogogues,........................ 651
Gonorrhœa and Syphilis, Treatment of,................................ 583
Gastralgia and Irritable Stomach, 414
Gum and Starch Paste, Preservation of,.................................. 303
Galvanism in Tetanus,............... 303
Gangrene in Hospitals,..........307, 674
Gall-bladder, Rupture of,............ 210
Gout, Rheumatism, Rheumatic Gout, &c.,............................. 230
Guy's Hospital, Presidency of,..... 118
Glycerine,................................ 58
Gilbert, H. F., on Bromine in Dysentery, &c.,.......................... 657

Hospital, for Small Pox in Chicago,....................................48, 300
Hæmostasis,............................. 61
Hospitals, French and English..... 121
Holderness, E., on Rupture of Uterus,.............................. 216
Human Physiology, Treatise on,... 244
Human Body, its Height, Weight, &c.,...................................... 249
Hydrophobia, Cure of,................ 345
Heart-Clot, as a Cause of Death in Diphtheria,........................... 349
Holmes, E. L., on Diseases of the Eye, &c.,.........................385, 651
Handmaiden, a Plea for,............ 509
Hammond, W. A., on Venerial Diseases, &c.,..............518, 587, 650
Hepatic Disease. By D. Trimble,... 538
Hypodermic Treatment of Uterine pain,...................................... 580

Insane Women, their Management, 626
Introductory Lecture. By N. S. Davis,................................... 616
Iodine Potassium in Asthma,....... 570
Illinois State Medical Society, Proceedings of, &c.,....107, 181, 247, 299
379, 368, 712, 713
Iridectory, New Mode of Operating for,.................................. 294
Iodine as a Deodoriser and Disinfectant, &c.,.......................54, 241
Infantile Mortality in New York,.. 252
Injections, Topical of Strychnine in Facial Paralysis,................ 123

Page.
Illegal Detention, Action for,...... 253

Jennings, J. D., on Diphtheria..... 273

Know. A Lecture by F. W. White, M.D.,.............................. 181

Langer, Ignatius, on Sub-Cutaneous Injections,..................413, 442
Lyman, H. M., on Abscess of the Gall-Bladder,........................ 210
Longevity, Extraordinary,......... 251
Liverpool, Mortality of,.............. 178

Malformations,.......................... 13
Medical Staff of England,......... 54
Mortality Statistics,..........108, 178, 252, 300, 652, 709
Metritis, Chronic,...................... 124
Medical Formulary,................... 179
Mercury as a Remedy,............... 242
Matchette, Aliquie C., Letter from, 281
Medical Independent, &c., of New York,..................................... 302
McVey, R. E., on Cerebro-Spinal Meningitis,............................. 403
Medical News,.....................443, 444
Medical Diagnosis, DaCosta on..... 513
Manual of Practice of Medicine,.. 513
Medical Register of New York. By Guido Furman, M.D.,.............. 513
Marriage, Philosophy of,............ 514
Movement Cure, Theory and Practice of,...................................... 514
Medical Journals, Suspension of,.. 517
Medical Colleges in Chicago,....... 517
Miller, DeLaskie, on Puerperal Fever,........................................ 529
Medical Association of Fox River Valley,..................................... 565
Medical Practice in Chicago, Profits of,........................................ 711

Neuralgic Affections, from Injuries, &c.,.............................. 636
Neuroma, Cure of,..................... 245
Nitrous Oxide as an Anæsthetic,... 78
Nervous Force, Velocity of,......... 33

Organs of Sensation and Motion,.. 1
Oxygen Gas,........................316, 716
Oxygennesis, Production of Pure Oxygen,................................... 362
Orthopedic Surgery, Report on,... 463
Obstetrics, Principles and Practice of,.......................................... 513
Orthopedic Surgery, Lectures on, 245

Page.
Opium and Quinia Sulpatis,......... 661

Pulmonary Emboli, in Contusions and Fractures,....................... 715
Promotion,................................. 587
Pregnancy, Vomiting in,............ 588
Paraphlegia,............................. 589
Practical Medicine, Report on, 321, 449
Prince, David, on Orthopedic Surgery,...................................... 463
Pharmacopœia, British,.........123, 252
Permanganate Potash,............... 154
Practical Pharmacy, Treatise on,.. 181
Professional Dignity in France,... 189
Perseverance,............................ 189
Powell, W. Byrd. Essay,.......... 1
Poisoning by Strychnine,............ 125
Pocket Surgical Cases,............... 129

Rush Medical College, Commencement,...................................... 107
Reid, John, on Catalysis,............ 132
Rheumatism, Tendinous treated by Sulphur,................................ 305
Rheumatism, Gout, and Sciatica, 516, 665
Report on Surgery,.................... 541

Sulphur, Test for,...................... 61
Sanitary Commission, U. S.,........ 51
Syphilis, Latent,........................ 44
Sarracenia Purpurea in Variola, 105, 107
State Board of Examiners for Degree of M.D.,............................ 111
Strychnine, Hypodermic Injections of,.................................. 123
Syphilis Conveyed by Vaccination, 125
Surgery, British, in Paris,............ 178
Stimulants in Fever, Use and Abuse of,........................................ 249
Structure of Indurated Chancre,... 309
Sub-Cutaneous Injection of Quinine,...................................... 413
Specialities,.............................. 428
Surgical Cases. By Dr. Amerman, 458
Surgeon General of New York,... 716
System of Surgery. By S. D. Gross, M.D.,...................................... 585
Surgical Diagnosis, Outlines of,.... 584

Tetanus, Traumatic, Treated with Chloroform, &c.,........................ 306
Terchloride of Carbon,............... 310
Tendo-Achillis, United by Silver Wire,....................................... 314
Therapeutics and Materia Medica, 650

Page.
Treatise on Chronic Inflammation, &c., of Uterus,........................ 514
Trimble, D. B., on Case of Hepatic Disease,................................ 538
Tobacco, Effects of,.........125, 697, 714

Uterine Action during Sleep,...317, 588
University of London,................ 243

Velpeau, M., at Academy of Medicine, .................................. 251
Vaccination, Report on,.........125, 257
Varicose Veins,......................... 308
Vaccination in the Army, Bad. By Dr. G. O. Smith,..................... 218

Page.
Varicocele of Twelve Years' Duration Cured,........................... 46

Wanzer, H., Cases in Practice,..... 21, 149, 410, 612
Wickersham, S., on Vaccination,.. 257
Whooping Cough, Belladonna in, 315
Wing, Henry, Valedictory Address,.......................................... 193

Young, D. W., on Cerebro-Spinal Meningitis, and Erysipelas,... 220, 405
Yager, E. Y., on " " 395

### BLISS & SHARP,

Wholesale & Retail

### DRUGGISTS AND CHEMISTS,

**144 LAKE STREET, CHICAGO,**

Keep constantly on hand a large assortment of

**PURE DRUGS FINE CHEMICALS,**

THAYER'S FLUID EXTRACTS & SUGAR-COATED PILLS

**Keith's Preparations,**

Agents for the sale of

**Tiemann's Celebrated Surgical Instruments,**

Trusses, Elastic Stockings, Bandages &c.

**Particular Attention Paid to Physicians' Orders.**

---

THE

### CHICAGO MEDICAL EXAMINER

N. S. DAVIS, M.D., EDITOR.

A MONTHLY JOURNAL

DEVOTED TO THE

EDUCATIONAL, SCIENTIFIC, AND PRACTICAL INTERESTS

OF THE

MEDICAL PROFESSION.

The EXAMINER will be issued during the first week of each month, commencing with January, 1860. Each number will contain 64 pages of reading matter, the greater part of which will be filled with such contents as will directly aid the practitioner in the daily practical duties of his profession.

To secure this object fully, we shall give, in each number, in addition to ordinary original articles, and selections on practical subjects, a faithful report of many of the more interesting cases presented at the Hospitals and College Cliniques. While aiming, however, to make the EXAMINER eminently practical, we shall not neglect either scientific, social, or educational interests, of the profession. It will not be the special organ of any one institution, society, or clique; but its columns will be open for well-written articles from any respectable member of the profession, on all topics legitimately within the domain of medical literature, science, and education.

Terms, $2.00 per annum, invariably in advance.

